# A Deep Learning-Based System (Microscan) for the Identification of Pollen Development Stages and Its Application to Obtaining Doubled Haploid Lines in Eggplant

**DOI:** 10.3390/biology9090272

**Published:** 2020-09-05

**Authors:** Edgar García-Fortea, Ana García-Pérez, Esther Gimeno-Páez, Alfredo Sánchez-Gimeno, Santiago Vilanova, Jaime Prohens, David Pastor-Calle

**Affiliations:** 1 Instituto Universitario de Conservación y Mejora de la Agrodiversidad Valenciana, Universitat Politècnica de València, Camí de Vera s/n, 46022 Valencia, Spain; angarpre@upv.es (A.G.-P.); esgipae@upv.es (E.G.-P.); sanvina@upvnet.upv.es (S.V.); jprohens@btc.upv.es (J.P.); 2 Seeds For Innovation, Calle Regaliz, 6, 04007 Almería, Spain; alfredo.sanchez@seeds4i.com; 3 SOMDATA, Carrer d’Alvaro de Bazan, 10, 46010 València, Spain; david.pastor@somdata.es

**Keywords:** androgenesis, anther culture, microspores, RetinaNet, *Solanum melongena*

## Abstract

The development of double haploids (DHs) is a straightforward path for obtaining pure lines but has multiple bottlenecks. Among them is the determination of the optimal stage of pollen induction for androgenesis. In this work, we developed Microscan, a deep learning-based system for the detection and recognition of the stages of pollen development. In a first experiment, the algorithm was developed adapting the RetinaNet predictive model using microspores of different eggplant accessions as samples. A mean average precision of 86.30% was obtained. In a second experiment, the anther range to be cultivated in vitro was determined in three eggplant genotypes by applying the Microscan system. Subsequently, they were cultivated following two different androgenesis protocols (Cb and E6). The response was only observed in the anther size range predicted by Microscan, obtaining the best results with the E6 protocol. The plants obtained were characterized by flow cytometry and with the Single Primer Enrichment Technology high-throughput genotyping platform, obtaining a high rate of confirmed haploid and double haploid plants. Microscan has been revealed as a tool for the high-throughput efficient analysis of microspore samples, as it has been exemplified in eggplant by providing an increase in the yield of DHs production.

## 1. Introduction

The development of commercial hybrids from elite materials and the introgression of genes of interest from related species that allow expanding the genetic background of crops [1] are basic tools for breeders [2]. Hybrids have multiple advantages, including heterosis for productive traits and they facilitate the accumulation in a single genotype of resistance to multiple diseases conferred by dominant genes [3]. To obtain genetically uniform F1 hybrid plants, the development of pure lines (highly homozygous organisms that breed true by selfing) is one of the main objectives of plant breeders. However, the development of pure lines is not simple and may require a long time [4]. 

The conventional method to obtain lines has traditionally been the application of successive rounds of self-fertilization and selection, generally requiring 7 to 10 generations for achieving a high degree of homozygosity. By reprogramming the gametophytic development pathways to sporophytic or embryogenic development pathways through the application of different inducing agents in microspores [5], pure lines can be obtained very quickly. This generally requires applying an appropriate stimulus or stress to pollen precursors to induce such reprogramming. These stresses can be very varied, including high or low temperatures, starvation, elicitors, or radiation, and they can be applied both to isolated microspores or to anthers [6,7,8]. However, this is not a straightforward and simple process and it must also be considered that there are many differences among species and among genotypes within species in the success of the induction. In many cases, small differences in the development stage of pollen translate into great changes in the efficiency of the application of the stresses for a successful induction. This is because not all the stages of the development of pollen are competent for a successful induction in the process of androgenesis. In general, the stages of vacuolated microspore and young pollen are those that will undergo reprogramming towards the embryogenic route in a more efficient way [9,10].

The induction of androgenesis is even more challenging in recalcitrant species, including a number of Solanaceae crops, such as eggplant (*Solanum melongena* L.) [11]. Therefore, it is necessary to develop methodologies that maximize the success in the process of inducing androgenesis and that simplify the process. In eggplant there are protocols for the cultivation of anthers and microspores in which both induction factors and culture media are very well defined [12,13]. However, the selection of the optimal stage for the induction of the androgenesis process is a bottleneck in the process and for which there has not been a practical and efficient solution to date. Thus, prior to the in vitro culture process for androgenesis induction, it is advisable to identify the size of the buds or the anthers with the highest proportion of vacuolated microspores or young pollen [14,15]. This is a fundamental step to ensure that the tissues that are being submitted to the used protocol are in an adequate stage of development and that the efficiency in androgenesis will be maximized. This generally requires the examination of multiple anthers and the manual counting of microspores in appropriate developmental stages [16]. Some general optical systems and software are known to provide an alternative to manual counts [17,18,19], which continue to be considered as the most reliable technique to date, at least in the case of microspore counting. However, pollen cell stages are partially determined by image details with a high degree of complexity that cannot be discerned with these techniques. In consequence, they have a significant identification error ratio.

The main issue with current automatized systems for the identification of the stages of development of pollen is the approach used. Generally, this is based solely on trying to parameterize, from the expert knowledge, the complexity of the image of a cell [20,21,22]. This approach has largely failed to date, because the number of variables that must be parameterized for this approach to work successfully is very large. For this reason, we present as an alternative the development of a model based on deep learning. These models are based on extracting statistical patterns from previously characterized pollen cells in different developmental stages [23]. With these patterns, a classifier model is fed to identify the position of the cells in the image and their stage of development. Specifically, the model used in this work consists of a convolutional neural network for image segmentation. These types of computer imaging models have evolved greatly in recent years, reaching for certain types of recognition, such as blood cells [24,25], retina cell nuclei [26], even COVID-19 diagnosis [27,28], with yields superior to those of humans [29].

The main objective of this work was to develop a deep learning-based system, named Microscan, which facilitates the selection of anthers in the appropriate stage of development for obtaining a high yield of doubled haploids through anther culture. Microscan consists of the combination of an automated microscope that scans the samples and an algorithm of artificial intelligence based on machine learning techniques that can make accurate predictions, at least as good as a trained technician, of the proportion of microspore cells in different stages in eggplant. In this way, through the use of Microscan, we were able to make the process more efficient in order to improve the success of androgenesis. For this, we used eggplant introgression materials for testing the efficiency of the approach proposed.

## 2. Materials and Methods

### 2.1. Plant Material

Three *S. melongena* accessions, namely MEL1, MEL 3 and MEL5, and two pre-breeding populations, consisting of a fourth backcross (INSBC4) generation of an introgression line between *S. melongena* MEL5 and *S. insanum* INS1, and a second selfing of a second backcross (MBC2S2) generation between *S. melongena* MEL1 and *S. anguivi* ANG1 were used for the algorithm training.

Three genotypes of one of the families of the introgression line between *S. melongena* MEL3 and *S. elaeagnifolium* ELE2 (BC3 17-8, BC3 17-19, BC3 17-4) were used for testing the algorithm efficiency using two different anther culture protocols (E6 and Cb). Finally, BC4 and BC3S1 generations together with the doubled haploid plants derived from accessions BC3 17-19 and BC3 17-4 were used for genotyping. All plants were grown in 10 L pots at Universitat Politècnica de València (UPV) glasshouses (GPS coordinates 39.482228, −0.337332) using coconut fiber substrate and fertigation.

### 2.2. Experimental Layout and Workflow

**Figure 1:**
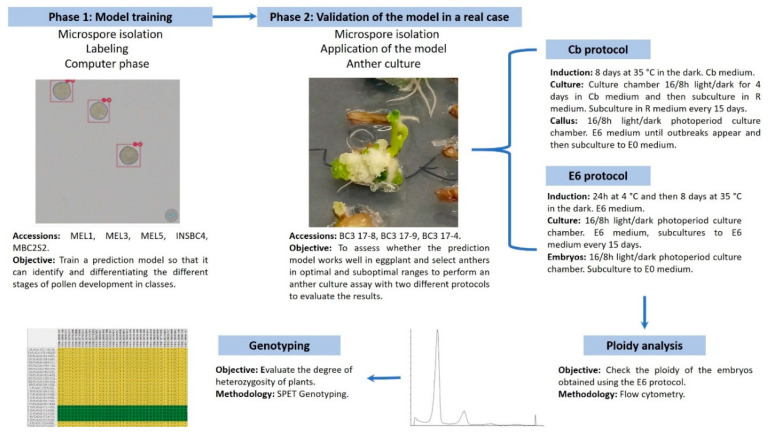
Description of the experimental design and the main objectives. The flow of the arrows indicates the order in which the experiments were conducted and their relationships.

The experimental layout and workflow are presented in Figure 1. Phase 1 was carried out to develop an instance segmentation model called RetinaNet [29] based on deep learning that could identify and classify the different stages of pollen development. This model needs to be fed with sample images similar to those that would be classified. For this, concentrated extracts of the different stages from tetrad to mature pollen were obtained from the different accessions of cultivated eggplant and experimental breeding populations. Cells were manually tagged in thousands of digitized images from these extracts. Then, images were split between a training set to feed the model and a validation set to evaluate the accuracy. The model parameters were tuned with the image information during the training phase, until the accuracy was enough to infer tags into new images [30].

The following phase of this experiment consisted of the validation of the model obtained in Phase 1. In Phase 2, the Microscan system was applied to a real case in eggplant. In this experiment, we tried to check if the level of androgenic response improved by selecting the anthers using the prediction model. For this, the anthers recommended by the model were selected and suboptimal ranges were selected as a negative control. These anthers were then cultivated using two different protocols, one of reference (Cb) [12] with some modifications and the another one newly developed in this work (E6). Finally, the plants obtained were analyzed by flow cytometry to ascertain its ploidy level and they were genotyped with the high throughput Single Primer Enrichment Technology eggplant platform [31].

### 2.3. Phase 1: Model Training

#### 2.3.1. Microspore and Pollen Isolation

In order to have enough cells at the same stage of development to facilitate the tagging task, synthetic samples were prepared by concentrating the cellular content of anthers with a range of similar size. For this, four size ranges of a 1 mm of eggplant anthers were arbitrarily established (<3 mm, 3–4 mm, 4–5 mm, and 5–6 mm) establishing a minimum size of 3 mm since it was not possible to work with smaller anthers and a maximum of more than 6 mm since most of the anthers of this size already contained pollen, and a pool was also prepared with the anthers of different size ranges, with microspores in all their stages, for testing the algorithm. To avoid biases due to possible morphological differences in cells due to differences among genotypes, samples from the eggplant accessions (MEL1, MEL 3, MEL5, INSBC4 and MBC2S2) were mixed. For each of the ranges, 20 flower buds which had at least five anthers each were harvested. That is, each cell concentrate came from a minimum of 100 anthers within the size range. The anthers of each rank were crushed in a beaker with the aid of a syringe plunger, followed by eluting with sterile distilled water. The solution was filtered with a 41 µm nylon membrane, then centrifuged with an ultracentrifuge refrigerated at 4 °C (Centrofriger-BL II, J.P. Selecta) at 850 rpm for 5 min. The supernatant was discarded, and the centrifugation was repeated twice. The microspores already free of tissue debris that could pass through the nylon membrane were resuspended in 5 mL of sterile distilled water, thus leaving the concentrate ready to acquire images with the Microscan system.

#### 2.3.2. Digital Data Image Acquisition

Twenty microliters of concentrated microspore solution was deposited on a slide and a 11 mm^2^ coverslip was placed on the top. In this way, a 0.165 mm water column was generated, high enough not to crush the cells and flat enough so that all the cells are at the same depth of field.

**Figure 2:**
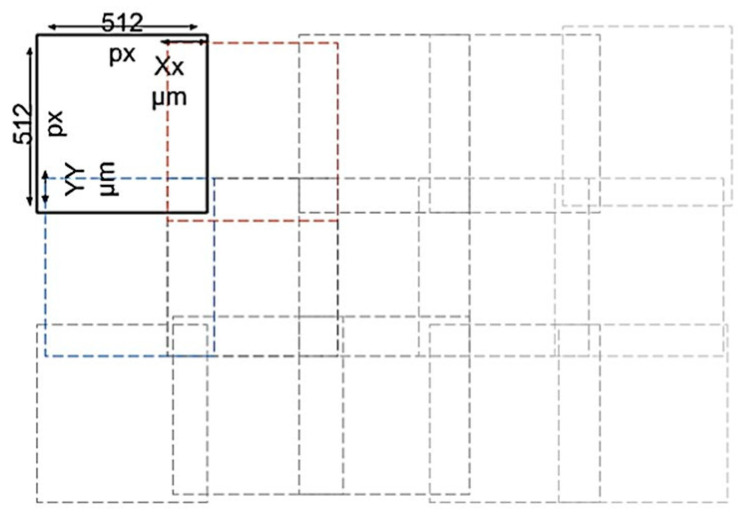
Scheme of how the imaging is performed using the motorized light microscope. The pictures are taken in a 512 × 512 px section, generating overlapping areas to obtain a macro image without blemishes in these areas.

The coverslip surface was scanned using an optical motorized digital microscope (MoticEasyScan One, Motic, Barcelona, Spain). It is a non-inverted brightfield optical microscope with a zenithal incoherent white LED illumination source (10W LED (Lifetime: 25,000 h)). To capture the images, an apochromatic l × 10/0.3 optical lens was used together with a zenithal camera with a resolution of 1 Mpx (2× digital zoom), in addition, the equipment incorporates another oblique camera for the detection of the focus plane (autofocus) with the same characteristics as the previous one. This microscope moves in a preselected area at regular steps, acquiring 512 × 512 px images corresponding to regions of a size of 0.26 mm^2^. The result is like the mosaic in Figure 2. To correct inaccuracies in the displacements due to the thread pitch, overlapping areas are used to algorithmically merge images by creating a macro image. The 512 × 512 px images were extracted from the generated macro image and used to train the deep learning model. For this, it was necessary to label them by an expert as well as to train the model.

#### 2.3.3. Image Labelling

**Figure 3:**
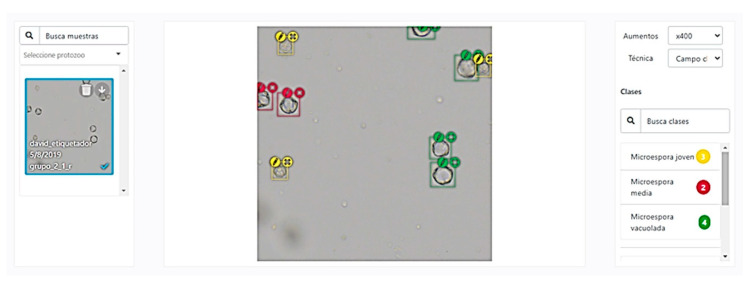
Graphic environment of the manual labelling software used for the classification of the microspores that was used for the Microscan model training. In the central part it can be seen how the cells have been labelled by an expert and classified according to their stage of development.

The chopped images from the previous section were inserted into a labelling software developed by the company SomData Analytics (Valencia, Spain) (Figure 3).

For the labelling of the images, a bounding box system was chosen which allows a balance to be struck between the quality of the labelling and the effort of the labeler. When a box system is used, all the structures present in the image must be labelled, although sometimes it may be unclear to which class some of the microspores belong to, therefore a stage defined as “doubtful” was added to the pollen development stages in order to revise the structure or to delete this image from the training.

**Figure 4:**
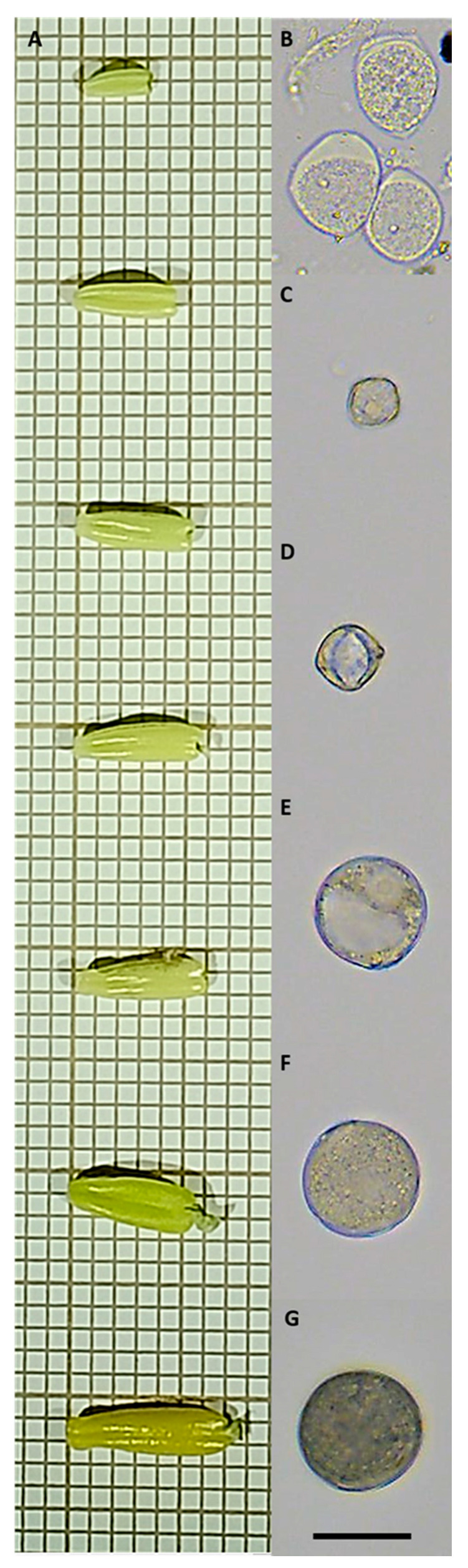
Eggplant anthers at different stages of development (**A**). Different stages of development of the pollen, tetrad (**B**), young microspore (**C**), medium microspore (**D**), vacuolated microspore (**E**), young pollen (**F**) and mature pollen stages (**G**). These have been the stages chosen to define the classes used to train the prediction model algorithm. The predominant stage of pollen development that each contained within each anther inside approximately corresponds to the state indicated in the image to the right of each anther size. The squares in the left images measure 1 mm, while the bar in the images in the right part of the figure measure 20 µm.

Six classes (tetrad, young microspore, medium microspore, vacuolated microspore, young pollen and mature pollen) were defined as different stages of development of the microspore that can be found inside the anthers depending on their size range (Figure 4A). The criterion used to define them was that they were easily differentiated by using an optical microscope, without the need for any type of histological or fluorescent staining. All these stages have different degrees of maturity and it is the criterion of an expert eye to define them. Even so, the model to be developed will work with a continuous distribution system with a confidence interval instead of a discrete distribution, allowing to assign a probability of belonging in different degrees to one class or another, thus solving the problem of intermediate stages. The eggplant tetrads have a very characteristic rhomboid-shaped morphology (Figure 4B); the young microspores have a low opacity and a reduced size compared to other stages (Figure 4C); the middle microspores (Figure 4D) have an intermediate size between the young and vacuolated microspores and the openings of the exine are clearly appreciated as more thick edges; vacuolated microspores have a more circular perimeter and in most cases a low cytoplasmic opacity where the vacuole itself is seen (Figure 4E); young pollen is larger than the vacuolated microspore and has a higher opacity due to the onset of the accumulation of starch granules (Figure 4F); mature pollen is the most opaque cell type due to the large accumulation of these granules (Figure 4G). Stages such as unicellular and bicellular young pollen could not be defined with this system due to the need to use 4′,6-diamidino-2-fenilindol for its classification [32].

A total of 2439 images were labeled and supervised by experts. A total of 290 tetrads, 643 young microspores, 1896 medium microspores, 1191 vacuolated microspores, 1876 young pollen and 2186 mature pollen cells were classified, making a total of 8082 manually classified cellular events.

#### 2.3.4. Preprocessing

The quality of the prediction model depends largely on the quality of the data with which it is fed. For this reason, it is necessary to pre-process the images to equalize the brightness and color so that their levels are similar in all the pictures, as well as to eliminate unfocused images, which are detected with Fourier analysis [33]. Furthermore, all the images in which there were no cellular structures were eliminated. 

The evaluation of the model skill on the training dataset would result in a biased score. Therefore, the model was evaluated with the typically called train–test split approach [34]. Specifically, the 2439 images were split into train, test and validation sets in proportions of 80%, 10%, and 10%, respectively. The subsets split maintained approximately the same imbalance ratio between classes than in the original set.

Another factor that must be considered and corrected is the imbalance. Due to the biological nature of the samples, it may occur that a cell group is overrepresented with respect to another, and this would make the prediction model assign by probability to the minority groups the class of the majority group. The way to solve this is to apply a weighted system, where the model pays more attention in its learning when cells of the minority group appear. This reduces the bias in which the model binds by the ruling class in the training set.

Other strategies to increase the quality of the prediction model including the use of image augmentation techniques. This means that the same images that are used to feed the model are modified by slightly rotating them, blurring or subtly altering the sizes. These same images are reintroduced to the model to be used in its training.

#### 2.3.5. Predictive Model

The predictive model used for the development of the Microscan is an adaptation of the RetinaNet instance detection network [29].

This model has been used since, in images where objects are sparse and most of the image is background, as it is one of the most efficient ones [29]. Instances are indicated with bounding boxes. Finally, the training and inference of these models is one of the fastest. In order to carry out this work, it was necessary to have a server with a GPU (graphic processing unit).

### 2.4. Phase 2: In Vitro Androgenesis Induction Test Using the Anther Selection Software

In this case, 8 anther size ranges were established (<3.5 mm, 3.5–4 mm, 4–4.5 mm, 4.5–5 mm, 5–5.5 mm, 5.5–6 mm, 6–6.5 mm, > 6.5 mm). Following the procedure described in the previous sections, the cell concentrates were prepared and subjected to the analysis of the prediction model.

Two different androgenesis protocols were applied, E6 (based on [35]) and Cb (based on [12]). For each of the three genotypes tested, three experimental replicates were performed with 15 anthers per replicate. Sizes were within the range determined by the algorithm, coinciding with that one which maximized the number of vacuolated microspores (5.5–6 mm), which were used for each of the two protocols. We also added three replicas with 15 anthers each below the recommended range (more specifically in the young microspore stage, 3.5–4 mm) and another three replicas with 15 anthers, each above the recommended range (more specifically, in the mature pollen stage, >6 mm). In total, 45 anthers were evaluated within the optimal range for each of the genotypes in both protocols (270 anthers) and a total of 45 anthers in suboptimal ranges as a negative control for each of the genotypes in both protocols (540 anthers).

#### 2.4.1. E6 Protocol

Firstly, the flower buds of the young plants were harvested during the autumn season of 2019 and a cold stress pre-treatment was applied, leaving them at 4 °C for 24 h. These buds and the anthers that were obtained were kept at 4 °C throughout the process. This disinfection of the buds, like the culture, was carried out in a laminar flow cabinet. First, the buds were immersed for 30 s in 70% ethanol, followed by 10 min in 20% commercial bleach with a few drops of Tween^®^ 20, and finally, three washings of 3 min each with constant agitation to ensure good sterilization were carried out with sterile distilled water. Once the buds were sterilized, they were left to dry for a few seconds on sterile filter paper. The anthers were extracted from inside the buds, measured, selected, and finally cultivated with their concave side down in medium E6 (Table 1) [35]. The plates were left at 35 °C for 8 days in dark conditions. After this induction period, the plates were taken to the culture chamber which maintained a temperature of 25 °C with a photoperiod of 16/8 h light/dark. A layer of filter paper was placed on the top to simulate diffuse light conditions. Thereafter, the anthers were subcultured in R medium (Table 1) every 15 days until the appearance of embryos, which, upon reaching the cotyledon stage, were subcultured in E0 medium (Table 1).

#### 2.4.2. Cb Protocol

The starting conditions are the same as those of the E6 protocol as regards the cultivation of the plant material and its collection. The protocol procedure is also identical to that described above and the anthers are cultured in the same way except that in this case the induction medium is Cb [12] (based on [36]) instead of E6 (Table 1). When the embryos appeared, they were subcultured in E0 medium (Table 1) (this is a modification of the original protocol in which the embryos are subcultured in V3 medium). In the event of the formation of calli, another modification was applied. These calluses were subcultured in E6 medium. If shoots were obtained, they would be subcultured in E0 medium.

#### 2.4.3. Flow Cytometry

**Figure 5:**
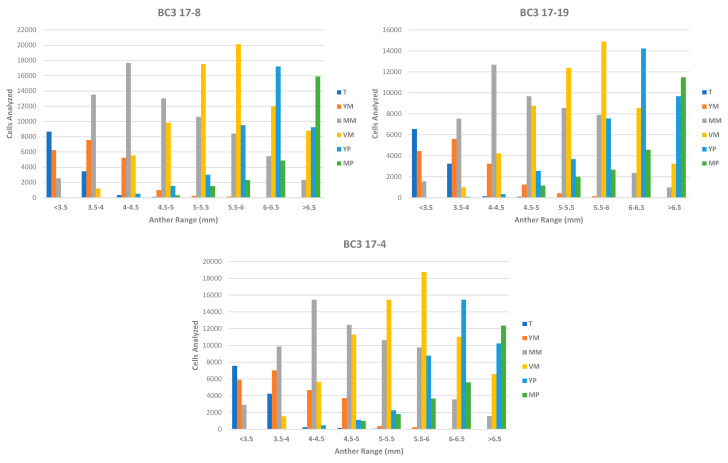
Distribution of the cell types identified by Microscan within each anther range in the three genotypes used for the Phase 2 experiments. The *Y* axis shows the number of cells classified for each type; the *X* axis shows the anther range corresponding to each analysis. The color code in the legend indicates the cell type, tetrad (T), young microspores (YM), medium microspores (MM), vacuolated microspores (VM), young pollen (YP) and mature pollen (MP).

Cell nuclei from leaf tissue were isolated mechanically according to Dpooležel [37] with modifications. Leaf sections of approximately 0.5 cm^2^ were chopped with a razor blade in a 6 cm diameter glass Petri dish containing 0.5 mL lysis buffer LB01 (pH 7.5) supplemented with 15 mM Tris (hydroxymethyl) aminomethane, 2 mM Na_2_EDTA and 0.5 mM spermine, and incubated for 5 min. Subsequently, the suspension containing nuclei and cell fragments was filtered through a 30 μm CellTrics filter (Sysmex, Sant Just Desvern, Spain). The nuclei in the filtrate were stained with CyStain UV Ploidy (Sysmex) and incubated for 5 min. The fluorescence intensity of the homogenate was measured using a CyFlow ploidy analyzer (Partec, Münster, Germany), measuring at least 4000 nuclei for each sample. Using young leaves of a diploid eggplant, the diploid control peak was established at 50 points of the arbitrary intensity value of the fluorescence in the histogram (Figure 5). By comparison with this peak, the ploidy of the other tissues evaluated was checked.

#### 2.4.4. Single Primer Enrichment Technology (SPET) Genotyping

Genomic DNA was isolated from 3–4 true leaves, according to the Cetyl Trimethyl Ammonium Bromide protocol [38] with slight modifications. The extracted DNA was dissolved in Milli-Q water and general quality was confirmed in agarose gel at 0.8%. After a concentration measurement using a Qubit 2.0 Fluorometer (Thermo Fisher Scientific, Waltham, MA, USA), the DNA was diluted at 30 ng/µL for SPET analysis. The Single Primer Enrichment Technology is a mass genotyping approach that is based on the PCR amplification of regions of the genome using primer libraries (previously developed from specific sequencing and resequencing works for the species in question) that are located around a single nucleotide polymorphism [31]. Using the Tassel software [39], a filter was applied in which only those positions for which the donor accessions were heterozygous were selected. Later this filter was applied to the derived materials (BC4, BC3S1 and double haploids (DHs)) to evaluate their percentage of heterozygosity.

## 3. Results

### 3.1. Phase 1: Model Training

The precision results of the prediction model developed in this work are presented in Table 2. The precision success percentages were in all cases higher than 80%, the highest being in the case of mature pollen where a value of 92.19% was reached, followed by the vacuolated microspores, which reached 87.60%. The lowest percentage of precision was found for the medium microspores (81.97%). The mean average precision (mAP), a metric for measuring the accuracy of object detectors, in the case of our model, reached an average value of 86.30%. These percentages are based on a very high number of samples analyzed (2439 images and 8071 cells labeled manually), giving great statistical robustness to the model. A simple application has also been developed to generate graphical reports where the analysis of the prediction model can be easily accessed.

Finally, the average number of cellular events inferred by the trained prediction model was 2000 every 10 min. This performance ratio was calculated based on an area of 11 mm^2^, which is what the coverslip measures, and for a concentrated cell solution. As mentioned in the Materials and Methods, the performance ratio is subject to the processing capacity of the equipment with which it works, achieving the best performance with equipment that has a GPU.

### 3.2. Phase 2: In Vitro Androgenesis Induction Test Using the Microscan

The prediction model was applied to three genotypes of interest from three BC3 generations of the introgression lines of eggplant with *S. elaeagnifolium*. These genotypes presented a theoretical degree of heterozygosity of 12.5% and might require several generations of selfing to be fixed in homozygosity. As an alternative to the successive generations of self-fertilization, it was decided to select these individuals for the prediction model validation experiment. Figure 5 shows the results of the Microscan analysis when applied to the eight anther size ranges selected for the execution of this second phase. After applying the prediction model, for these genotypes, the size range of the anther that maximized the number of cells in the vacuolated microspore stage was determined to be of 5.5–6 mm. The mixture of anthers of this range presented a number of vacuolated micropores of 20,115 for genotype BC3 17-8, 14,887 for genotype BC3 17-19 and 18,775 for genotype BC3 17-4, which in the three cases was the highest of all those found in all defined anther ranges. On the other hand, the ranges for the control with young microspores and mature pollen were 3.5–4 mm and >6.5 mm successively, being the ones that maximize these cell stages.

**Figure 6:**
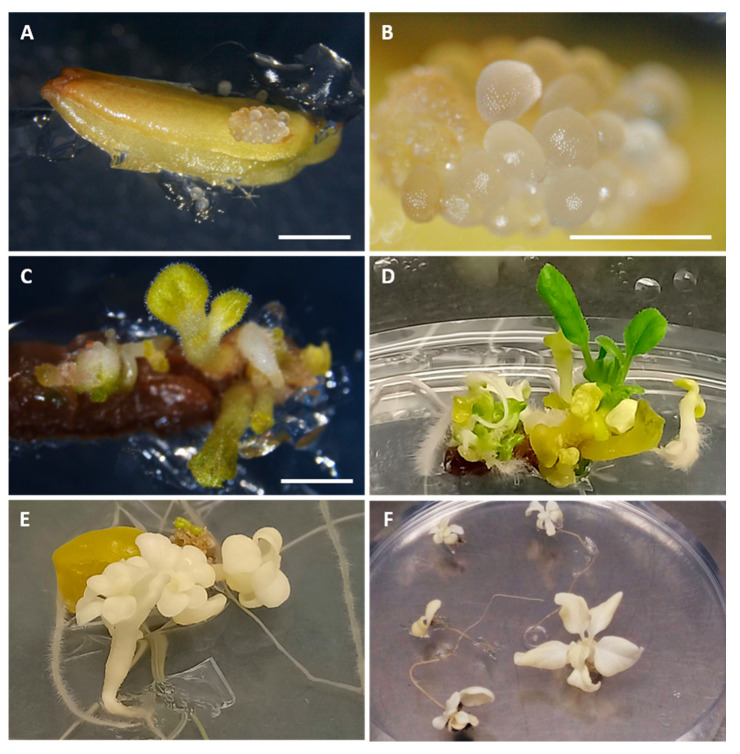
Anther selected through Microscan grown in E6 medium four days after being placed in culture (**A**); detail of the same anther, the formation of globular-type embryogenic structures can be observed (**B**); anther cultivated in E6 medium after one month of culture, it can be seen how embryogenic structures evolve giving rise to torpedo embryo-like structures and in some cases cotyledonal stage (**C**); fully developed seedlings and cotyledonal embryos of eggplant after one and a half months of culture in E6 medium (**D**); albino eggplant embryos from anther culture in E6 medium (**E**); albino embryos isolated from the anthers and subcultured in E0 base medium (**F**). The size of the bars is 1 mm.

Between 40 and 70% of the anthers of the range with vacuolated microspores (5.5–6 mm) subjected to the E6 protocol presented an embryogenic type response in the three genotypes evaluated (Table 3). On the other hand, the anthers of the negative control ranges did not show any type of response (Table 3); they were necrotic and did not give rise to any type of embryogenic or organogenic structure. After 4 days of induction at 35 °C, structures like globular embryos began to be observed (Figure 6A,B) in the 5.5–6 mm range. After a month of culture, these structures evolved giving rise to torpedo and cotyledon-type embryos (Figure 6C) which after a month and a half of anther culture already had a seedling appearance (Figure 6D) capable of rooting and being subcultured in an E0 medium to allow its development and subsequent acclimatization. In some cases, abnormal albino embryos also appeared that were completely unviable (Figure 6E,F). The average embryogenic structures per induced anther for the three genotypes had a similar value of around 4.

Table 4 shows the results of the total number of normal embryos generated in the anthers of the range predicted as the most efficient by the model. It also shows the number of plants acclimatized from these embryos and their ploidy. In genotype BC3 17-8, a total number of 42 embryos was obtained; of these, only 12 came to give acclimatized plants that were mostly haploid and some of them were mixoploid. In the case of genotype BC3 17-19, the number of total embryos was 29 while that of acclimatized plants was 12, and in this case the cytometric analysis showed that eight of these plants were haploid, one myxoploid and three diploids. Finally, the genotype BC3 17-4 generated nine embryos, seven of which gave rise to acclimatized plants, of which six were haploid and one was mixoploid. 

**Figure 7:**
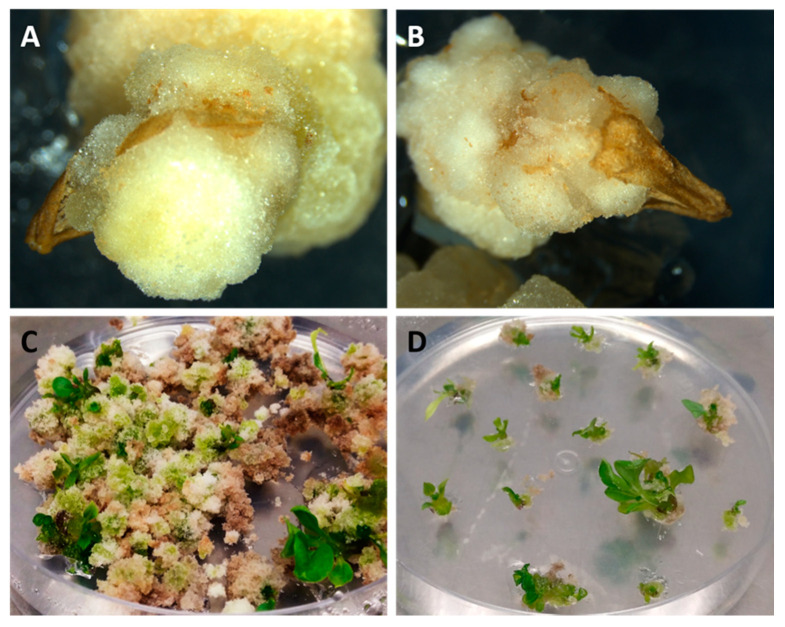
Calluses obtained two months after the culture of anthers in medium R applying the Cb protocol (**A**,**B**). By subculturing these calluses in E6 medium, shoot induction was achieved after approximately one month of induction in this medium (**C**) and finally the seedlings can be isolated (**D**).

Anthers within the range recommended by the prediction model (5.5–6 mm) showed androgenic response in Cb protocol in the three genotypes evaluated. As in the previous case, none of the negative control ranges gave rise to androgenic response, generating premature necrosis in both ranges. In the range of >6 mm, a response in around 4% of the anthers was observed too, and some somatic calli were formed in the cutting area where the filament was (Table 3). In the anthers of 5.5–6 mm, the formation of embryogenic structures did not occur, instead the formation of androgenic calluses that came from inside the anther was observed in 70–80% of the cases (Table 3), the calli burst the wall and emerged from within the anther (Figure 7A,B) after approximately two months of culture (i.e., one month after subculturing to medium R). Plants could be obtained by subculturing these calluses in E6 medium and subcultured every 15 days until the appearance of shoots (Figure 7C), which took place between one month and two months after subculturing the calluses in E6 medium for the first time. Finally, the seedlings obtained were subcultured to E0 medium (Figure 7D).

The cytometry data was contrasted with a Single Primer Enrichment Technology (SPET) high throughput genotyping, in which 532 single nucleotide polymorphism (SNP) markers were evaluated for which the anther donor plants (BC3 17-19 and BC3 17-4) were heterozygous (Table 5). Apart from genotyping the donor plants, individuals of the BC4 generation obtained by backcrossing between the donor plants and their cultivated parent (MEL3) were genotyped. There were six in the case of plant BC3 17-19 and five in the case of the plant BC3 17-4. It should be noted that during the growth process, many of the haploid plants showed spontaneous duplication events, and it was these that were selected for genotyping together with the accessions that were myxoploid and diploid in the first cytometric analysis. Ten of the 12 acclimatized plants of putative androgenic origin of plant BC3 17-19 were genotyped as well as all acclimatized plants (7) of plant BC3 17-4. The donor plants have a percentage of heterozygosity of 100% since during the analysis of the data, only those positions in which these plants were heterozygous were selected to filter the rest of the SNP positions in the accessions derived from this. In the analysis, for the BC3 17-19 accession, its offspring obtained by backcrossing (BC4) displayed a percentage of heterozygosity with an average of 55.07% (range of 39.06–65.66%). On the other hand, all DH individuals derived from this accession have reduced their heterozygosity with an average of 1.59% (range 0.19–3.95%). Very similar results are found in the case of accession BC3 17-4, where individuals obtained by backcrossing have high heterozygosity values with an average of 53.87% (range 13.35–92.66%), whereas here DH individuals have an average of 0.67% of heterozygosity (range 0.19–1.70%).

## 4. Discussion

The determination of the optimal stage for the induction of androgenesis is highly relevant for the development of doubled haploids, especially in recalcitrant crops such as eggplant. For this reason, the development of a tool like Microscan represents a great technical advance around double haploid production and in the study of androgenesis.

The model developed using a deep learning approach has proved highly efficient for the identification of the stages of pollen development. In this way, the mean average precision of the model is within the appropriate values for this type of forecasting system [23,24]. For the specific doubled haploids’ development application, a vacuolated microspore detection model would have been sufficient. The vacuolated microspore stage is the factor that will determine which range of anther sizes to select, as this is the most inducible stage in the androgenesis processes [14,15]. The reason for choosing the length of the anther as a morphological marker was that, unlike other species such as pepper where the anthocyanin pigmentation of the anthers is quite common, in our case the genotypes of eggplant that we used do not present this pigmentation, which means that the size of the anther seemed to us a more robust parameter and less dependent on genetic or environmental factors [11]. However, discriminating among stages makes the estimation of the optimal stage more reliable since it is not judged on a single stage, but on a distribution of stages. Just as an expert eye can differentiate different degrees of maturity in the development of a cell type, the algorithm used here is able to assign a confidence interval around each of the predictions for each of the cells. This generates a distribution that allows having a more accurate interpretation of the content of a specific anther. Regarding the specific precision values in some cell types, it should be noted that the lowest values correspond to those intermediate stages that are more difficult to differentiate, such as the case of medium microspores or young pollen, although even in this case, the correct identification values are over 80% [23,24]. On many occasions, the morphology of these cells is intermediate between the young and vacuolated microspore stages in the case of the medium microspore, or vacuolated and mature pollen in the case of young pollen. There are a large number of cellular events that are located halfway between stages that are very well established and recognizable (as is the case of young microspores, vacuolated microspores or mature pollen) and this is what makes the model more hesitant to assign those classes in those cases. Therefore, we decided to establish a continuous distribution system with a confidence interval instead of a discrete distribution. Just the opposite has happened with the tetrads, which despite being the class with the least number of cellular events tagged, has been one of the classes with the higher percentage of precision (87.40%) due to its unique shape and completely different from the rest of cells. The prediction model has obtained a very satisfactory level of precision that, added to its capacity for cellular analysis per unit of time and the large sample size that it is capable of handling, not only rivals the classification made by an expert technician in cataloging accuracy, but it also vastly outperforms it in volume analysis capabilities [40,41]. It should be remembered that these tasks when performed manually are highly time consuming and visually exhausting [42].

The use of the prediction model has been decisive in selecting those anthers that have shown the best induction results in the genotypes BC3 17-8, BC3 17-19, BC3 17-4, used during Phase 2. The selection of anthers with a high content of vacuolated microspores has been the only one to give an androgenic response, since this stage, together with the stage of young bicellular pollen, is the most inducible [14,15] and in this case, the algorithm maximizes that cellular percentage, adjusting the range of the anthers to be selected very well. Androgenic response has not been observed in those ranges associated with a cellular content of young microspores or mature pollen, non-inducible stages by default and by excess with respect to the optimal stage (vacuolated microspore/young bi-cellular pollen). Some authors suggest that due to the fact that the transcriptional state of the cell is still proliferative and it is not completely differentiated in the case of optimal stages [43], since at the moment in which the grains of pollen begin to accumulate starch, lose their embryogenic capacity, giving rise to the gametophytic development pathway [44,45]. Although this was expected, the fact of being able to compare these results with those obtained in the optimal range gives strength to the usefulness of the prediction model that has been developed. Finally, as we have seen, this result has been consistent and independent of the induction protocol and genotypes used, although the response observed has been different in the two protocols tested.

In the case of the E6 protocol, a direct embryogenic response was obtained. This effect could be associated with the use of ZR as a growth regulator during induction as seen in other works [46]. Furthermore, the appearance of albino embryos was observed, this being the first report in the anther culture of eggplant as far as we know, but which has been associated with androgenic events in other crops such as cereals [47,48,49,50,51], tobacco or arabidopsis [52]. On the other hand, the results observed in the Cb protocol are coincident with some observations by other authors in which, depending on the eggplant genotype used, they obtain embryogenesis or different types of callogenesis in this induction medium [16]. In our case, callus was obtained in all three genotypes using Cb medium, while those same genotypes in the E6 medium gave rise to embryos. 

The number of normal final embryos obtained using the E6 protocol was 77 among the three genotypes. Approximately 40% (31) of these embryos successfully acclimatized, giving rise to plants that, after being analyzed with flow cytometry, were mostly haploids (74.2% (23)), 9.7% (3) were diploids, and the remaining 16.1% (5) were mixoploids. The fact that most individuals were haploid ensures the androgenic origin of our materials and therefore the good result of the prediction model when choosing the correct induction stage. However, haploid plants present serious problems since they give rise to small flowers with different degrees of premature abscission, the abnormal formation of buds, anther and other organs within the flower and finally a reduction in the pollen viability [53,54,55,56], although in most cases spontaneous genome duplication is observed in this work, except for two individuals derived from accession BC 17-19, the rest of haploid plants ended up duplicating their genome spontaneously. For this reason, in these cases it is crucial to accompany the flow cytometric analysis with a molecular analysis; something that in some works has been done using sequence-related amplified polymorphism, simple sequence repeats or the random amplification of polymorphic DNA markers [57,58,59], but in many other works this is not done. Due to the dominance problems that some of the commonly used molecular markers have and the low resolution regarding the percentage of genome explored with them [60], we used high-throughput genotyping using the SPET technology [31] with over 500 single-nucleotide polymorphism (SNP) markers. The genotyping results have been conclusive, showing that in the majority of cases, the percentage of heterozygosity of individuals of supposed androgenic origin did not exceed 1%, although in some cases it was slightly more elevated, but did not exceed 5%. In another eggplant study, in which SNP-type markers were used, similar results were observed in plants from anther culture [61]. The appearance of a residual degree of heterozygosity in doubled haploids may be due to the effect of somaclonal variation during in vitro culture, or most likely as a consequence of the mapping of the SNP markers to paralog genes within the genome [61]. Therefore, our molecular results together with the cytometry results confirm the haploid origin of these materials.

The application of Microscan to a real case in eggplant has shown that it has been able to optimize a procedure to produce doubled haploids in this crop, automating one of the most tedious parts of the process. It has also made it possible to greatly reduce the time needed for the fine-tuning of the protocol in new genotypes. This represents an important saving in resources for determining the size of the anthers that contain the optimal microspore stage for the induction of androgenesis. The implications of Microscan in the field of plant genetic breeding and commercial seed production are therefore very important. In this way, having a more efficient method of producing doubled haploid lines also means having a powerful tool for conducting research in plant genetics and breeding. Thanks to their genetic stability and homogeneity, doubled haploids are excellent materials to carry out research because they do not have dominance or intra-family segregation effects [62]. For this reason, the studies of the association of molecular markers with phenotypic characters through the use of bulked segregant analysis [63], the selection of recessive mutants in Tilling experiments [64,65] or its use in genetic transformation experiments to avoid the appearance of hemizygous individuals [66], are just some examples of the great utility of double haploids, and therefore of how the Microscan indirectly represents an improvement in this area. Another approach that benefits from the use of the Microscan and that has already been presented as an example in this work, is the development of pre-breeding populations such as introgression lines. These populations help to greatly expand the genetic pool of cultivated species by incorporating introgressions from the related wild species [1]. In this case, the doubled haploid technique can accelerate the fixation process of said materials with introgressions, reducing the multiple self-fertilization cycles to a single generation and facilitating the genetic description of traits of interest. On the other hand, commercial hybrids bring great economic benefits to both the seed companies that produce them and the farmers who grow them. Heterosis confers a series of general favorable characteristics such as greater tolerance to biotic and abiotic stresses, phenotypic homogeneity or higher productivity and yield, which provide them with a high added value [67]. However, the production of hybrid seed depends on obtaining pure parental lines, this being the basis of the breeding pipelines of most seed companies. This represents a very important investment in the production of double haploid plants. The integration of Microscan to the double haploid production protocols of these seed companies can lead to a reduction in the production costs of pure parental lines, thus also representing a reduction in the cost of hybrid seed. Finally, the optimization of this system in other crops beyond eggplant would provide these same benefits for their breeding.

## 5. Conclusions

In this multidisciplinary study, we developed a system (Microscan) based on deep learning to overcome one of the bottlenecks in the production of doubled haploids (DHs) in recalcitrant species. The development of artificial intelligence systems represents a great advance to speed up light microscopy studies and, in this case, it has been demonstrated. The application of Microscan to eggplant genotypes has allowed selecting the optimal stage of induction which, together with the application of an innovative in vitro culture protocol, has allowed us to obtain DH plants with an androgenic induction rate higher than standard protocols and obtained excellent direct embryogenesis results. It is also the first time that this result has been confirmed with high-throughput genotyping in the case of eggplant. Our challenge now is to expand the detection capacity of Microscan to other species of commercial interest, thus increasing efficiency and breaking the barriers that in many cases are found when applying androgenesis techniques in many crops. Furthermore, this work can serve as a guide to develop similar applications where microscopy is used in agriculture and biology.

## Figures and Tables

**Table 1:** Composition of the different culture media used in the androgenesis protocols of Phase 2. The table collects the information on concentrations of basal salts, carbon source, growth regulators and gelling agent for the preparation of media E0, E6, Cb and R. Preparations C and R are specific formulations previously described [12,36].

Medium	MS + Vitamins (g/L)	Prepared C (g/L)	Prepared R (g/L)	Sucrose (g/L)	Zeatin Riboside (mg/L)	Kinetin (mg/L)	2,4-D (mg/L)	Vitamin B12 (mg/L)	Gelrite (g/L)	Bacto-Agar (g/L)
E0	2.20	-	-	15.00	-	-	-	-	7.00	-
E6	2.20	-	-	15.00	2.00	-	-	-	7.00	-
Cb	-	4.55	-	120.00	-	5.00	5.00	0.20	-	8.00
R	-	-	4.55	30.00	-	0.10	-	-	-	8.00

**Table 2:** Results of the precision in the model prediction for the different cell classes defined during the development of the eggplant microspore towards pollen grain. The mean average precision (mAP) of the algorithm is also presented.

Class	Total Cellular Events	Average Precision (%)
Tetrad	290	87.40
Young Microspore	641	86.28
Medium Microspore	1893	81.97
Vacuolated Microspore	1185	87.60
Young Pollen	1876	82.32
Mature Pollen	2186	92.19
mAP		86.30

**Table 3:** Percentage and type of response in the three eggplant genotypes tested for the two protocols (E6 and Cb) and the three anther ranges selected. The response percentage (±SE) is presented. The type of response has been differentiated depending on whether it was an embryogenic or a callogenic response.

	Protocol E6			Protocol Cb		
Size Range/Genotype	Anthers (n)	Response (%)	Type of Response	Anthers (n)	Response (%)	Type of Response
**3.5–4 mm**						
BC3 17-8	45	0.00 ± 0.00	-	45	0.00 ± 0.00	-
BC3 17-19	45	0.00 ± 0.00	-	45	0.00 ± 0.00	-
BC3 17-4	45	0.00 ± 0.00	-	45	0.00 ± 0.00	-
**5.5–6 mm**						
BC3 17-8	45	75.30 ± 0.04	Embryo	45	78.40 ± 0.04	Callus
BC3 17-19	45	60.40 ± 0.05	Embryo	45	80.50 ± 0.04	Callus
BC3 17-4	45	40.30 ± 0.05	Embryo	45	71.20 ± 0.05	Callus
**>6 mm**						
BC3 17-8	45	0.00 ± 0.00	-	45	3.20 ± 0.02	Somatic callus
BC3 17-19	45	0.00 ± 0.00	-	45	4.00 ± 0.02	Somatic callus
BC3 17-4	45	0.00 ± 0.00	-	45	4.30 ± 0.03	Somatic callus

**Table 4:** Total embryos obtained in the three genotypes of the BC3 family 17 from the culture of anthers of the 5.5–6 mm range using the E6 protocol. The total number of acclimatized plants and their ploidy level analyzed by flow cytometry are shown.

Genotype	Embryos (*n*)	Acclimatized Plants (*n*)	*n*	*n* + 2n	2*n*
BC3 17-8	42	12	9	3	0
BC3 17-19	26	12	8	1	3
BC3 17-4	9	7	6	1	0

**Table 5:** SPET mass genotyping results shown as a percentage of heterozygosity (mean and range) calculated based on the genotyping of 532 single nucleotide polymorphism (SNP) positions in the accessions derived from materials BC3 17-19 and BC3 17-4. Among these materials, we find backcrossings with the cultivated parental MEL3 (BC4) and plants obtained from the application of the E6 (DH) protocol. The number of positions SNPs evaluated and those that were not found in the specific accessions are shown.

Accession/Offspring	*n*	Missing SNPs	Heterozygosity (%)
ELE BC3 17-19	1	0.00	100.00
BC4 (ELE BC3 17-19 × MEL3)	6	4.00 (0.00–6.00)	55.07 (39.06–65.66)
DH (ELE BC3 17-19 doubled haploids)	10	5.00 (0.00–12.00)	1.59 (0.19–3.95)
ELE BC3 17-4	1	0.00	100.00
BC4 (ELE BC3 17-4 × MEL3)	5	0.40 (0.00–2.00)	53.87 (13.35–92.66)
DH (ELE BC3 17-4 doubled haploids)	7	1.14 (0.00–4.00)	0.67 (0.19–1.70)
